# Bioarchaeological and palaeogenomic portrait of two Pompeians that died during the eruption of Vesuvius in 79 AD

**DOI:** 10.1038/s41598-022-10899-1

**Published:** 2022-05-26

**Authors:** Gabriele Scorrano, Serena Viva, Thomaz Pinotti, Pier Francesco Fabbri, Olga Rickards, Fabio Macciardi

**Affiliations:** 1 Centre of Molecular Anthropology for Ancient DNA Studies, Department of Biology, University of Rome “Tor Vergata”, 00133 Rome, Italy; 2 Lundbeck Foundation GeoGenetics Centre, Globe Institute, University of Copenhagen, Copenhagen, Denmark; 3 Department of Cultural Heritage, University of Salento, 73100 Lecce, Italy; 4 Laboratório de Biodiversidade e Evolução Molecular (LBEM), Universidade Federal de Minas Gerais, Belo Horizonte, Brazil; 5 Laboratory of Molecular Psychiatry, Department of Psychiatry and Human Behavior, University of California, Irvine, CA 92868 USA

**Keywords:** Genetics, Population genetics

## Abstract

The archaeological site of Pompeii is one of the 54 UNESCO World Heritage sites in Italy, thanks to its uniqueness: the town was completely destroyed and buried by a Vesuvius’ eruption in 79 AD. In this work, we present a multidisciplinary approach with bioarchaeological and palaeogenomic analyses of two Pompeian human remains from the *Casa del Fabbro*. We have been able to characterize the genetic profile of the first Pompeian’ genome, which has strong affinities with the surrounding central Italian population from the Roman Imperial Age. Our findings suggest that, despite the extensive connection between Rome and other Mediterranean populations, a noticeable degree of genetic homogeneity exists in the Italian peninsula at that time. Moreover, palaeopathological analyses identified the presence of spinal tuberculosis and we further investigated the presence of ancient DNA from *Mycobacterium tuberculosis*. In conclusion, our study demonstrates the power of a combined approach to investigate ancient humans and confirms the possibility to retrieve ancient DNA from Pompeii human remains. Our initial findings provide a foundation to promote an intensive and extensive paleogenetic analysis in order to reconstruct the genetic history of population from Pompeii, a unique archaeological site.

## Introduction

**Figure 1:**
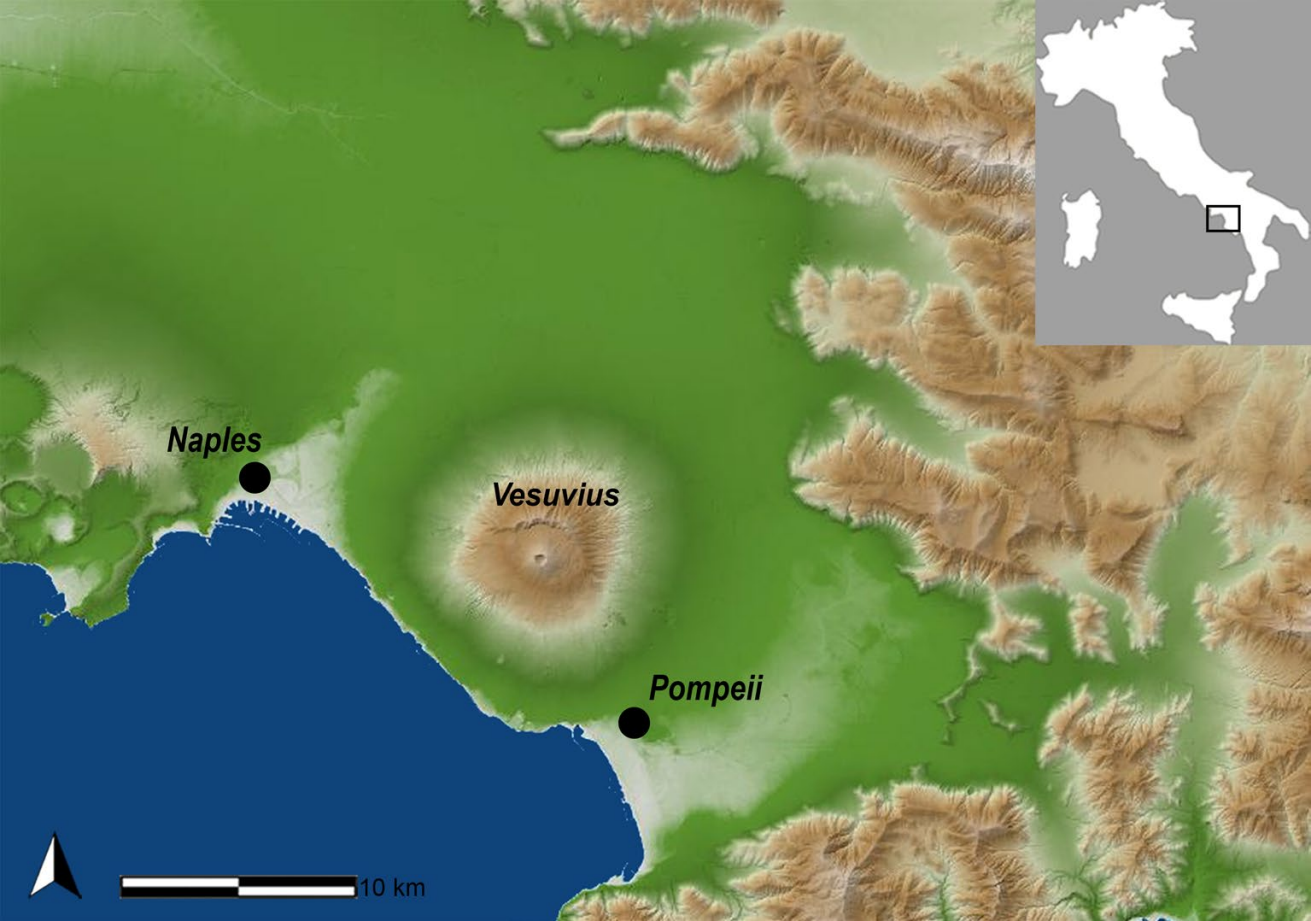
Geographic location of the Pompeii site, Campania (Italy). Map source: SINAnet ISPRA – Dem75 (QGIS 3.22 ‘Biatowieza’) https://www.qgis.org/it/site/.

The archaeological site of Pompeii is one of the 54 UNESCO World Heritage sites in Italy. Pompeii was a Roman Imperial Age port city located south of Naples in Central Italy (Fig. 1) until it was completely destroyed and buried by the ashes of the Mount Vesuvius’ eruption in 79 AD^1,2^. According to Gaius Plinius Caecilius Secundus (better known as Pliny the Younger: a lawyer, author, and magistrate of Ancient Rome), the Vesuvius’ eruption occurred around 1 p.m on the 24th of August and was visible from over 40 km away. More than 2000 individuals died as a direct consequence of the eruption^1^, the deadliest ever in European history. The several exceptionally well-preserved buildings found in Pompeii such as *Casa del Chirurgo* (House of the Surgeon), *Casa del Fauno* (House of Faun) and the *Casa dei Casti Amanti* (House of the Chaste Lovers) suggest that Pompeii was probably a holiday resort for wealthy Romans. However, Pompeii was also an important city for trading and business, with a population ranging between 6400 and 20,000 dwellers.

Despite continuing an intense scientific research on the site since the nineteenth century to this day, conducting both bioarchaeological and genetic studies from Pompeiian human remains has been a challenge, as exposure to high temperature effectively destroys the bone matrix, altering the structure of the bioapatite^3^ and diminishing the quality and quantity of recoverable DNA^4^. On the other hand, it is also possible that the pyroclastic materials that covered the remains could have shielded them from environmental factors, like the atmospheric oxygen, that degrades DNA^5^.

Past studies have shown the possibility of retrieving genetic data from both human and zooarchaeological remains in Pompeii^6–12^, but those initial analyses were limited to short stretches of mitochondrial DNA obtained using PCR-based methods. New available methodologies, based on high-throughput shotgun sequencing, DNA capture, enrichment strategy^13,14^, as well as using optimal sources of ancient DNA (aDNA) from teeth and petrous bones^15,16^, have dramatically increased the amount of data that can be obtained from previously unsuitable samples for genetic research, and may open new avenues to substantially increase the knowledge of the genetic diversity in the ancient Pompeian population.

In this work, we present a multidisciplinary approach with bioarchaeological and palaeogenomic analyses of two human remains from the *Casa del Fabbro* (House of the Craftsman: Supplementary Fig. S1) from Pompeii. The successful recovery of aDNA from one individual enabled us to reconstruct its genetic history and to investigate the presence of blood-borne pathogens, alongside skeletal biology evidence. Furthermore, this data can also give us an overview of the genetic diversity outside of Rome during the Roman Empire.

### Individuals analysed from Casa del Fabbro

The analysed human remains came from Room 9 of the *Casa del Fabbro* (Regio I, Insula 10, civic 7), and their position and orientation are compatible with instantaneous death due to the approach of the high-temperature volcanic ash cloud^17^. More than half of individuals found in Pompeii died inside their houses, indicating a collective unawareness of the possibility of a volcanic eruption or that the risk was downplayed due to the relatively common land tremors in the region^2^. Both skeletons have been discovered in anatomical position. They were both leaning on a low relief in a corner of what probably was the dining room, on the remnants of a *triclinium*, a sort of couch or *chaise longue* used in Roman buildings during meals. Individual A was in left lateral recumbent position with flexed limbs, with the left arm and leg on the ground and right limbs on the *triclinium*. The individual B had the arms gathered in front of the skull and legs on the ground flexed on the right side, with the back leaning against the *triclinium*.

## Results

The two *Casa del Fabbro* individuals (Supplementary Fig. S1) underwent osteological examination to establish their respective sex, estimated height, and approximate age-at-death. Individual A was a male between 35 and 40 years-old and stood 164.3 cm tall. Individual B was a female over 50 years of age who stood 153.1 cm tall. Estimated heights were obtained through averaging two methods^18–21^ and are consistent with Roman Age height averages (male: 164.4 cm; female: 152.1 cm)^22^ as well as Pompeii and Herculaneum height averages^23,24^ (see Supplementary Table S1).

We extracted and sequenced DNA from a petrous bone from each Pompeian individual with identical procedures (see “Materials and methods”) and obtained 0.4 X (individual A) and 0.0013 X (individual B) average genome-wide depth of coverage (Table 1). Both individuals displayed typical signatures of aDNA^25^ (Supplementary Fig. S2). Low rates of contamination with modern human DNA (0.8% for the mtDNA and between 0.6 and 0.8% for the X chromosome, Table 1 and Supplementary Figs. S2, S3) for individual A confirmed the authenticity of the aDNA. The low coverage obtained for individual B prevented us from reaching any further assessments of quality parameters. Herein, we report the details of the analyses only for individual A.

**Table 1:** Statistic parameters and contamination results of the individuals analysed. Total is the total number of reads per library; Unique is the number of sequences mapping uniquely to the human reference; Rmdup is the unique sequences without the duplicate; Endogenous (%) is the proportion of human sequences after trimming; genome-wide; we also reported the number of identified SNPs, the mitochondrial, X and Y-chromosome average depth of coverage.

Individuals #	Sample code #	Total	Uniq	Rmdup	Clonality%	Endo%	Coverage	Number of identified SNPs	mtDNA coverage	mtDNA haplogroup	Y-chromosome coverage	Y-chromosome haplogroup	mtDNA contamination (%)	X coverage	X chromosome contamination (%)	Sex determination
Pompei casa del Fabbro individual_A	f_1R	67,481,814	22,071,517	17,340,415	21.43	37.40	0.42	450.751	41.42	HV0a (85.3%)	0.074	A-M13	0,8 (15.3; 0.2)	0.25	0.8–0.6	XY
Pompei casa del Fabbro individual_B	f_11R	156,203,139	42,007	40,123	4.48	0.017	0.0013	/	/	/	/	/	/	/	/	/

### Sex determination and uniparental genetic markers

The genetic sex determination (estimated by R_Y_ parameter^26^, and by the X chromosome coverage) confirmed the morphological determination that individual A was a male (Table 1).

The mitochondrial DNA haplogroup was identified using HaploGrep2^27,28^ (Table 1), and revealed that the individual belongs to the haplogroup clade HV0a, the main monophyletic branch of HV0 and subclade of haplogroup HV. This mitochondrial lineage is absent among published Roman Imperial individuals from Italy^29^. In Europe, the first evidence of the HV haplogroup is from a Magdalenian period individual from Spain^30^ while in Italy from a Mesolithic individual from Sicily (Favignana)^31^. The HV haplogroup is actually associated with the early human dispersal in Eurasia after the Last Glacial Maximum (LGM)^32^. It is unevenly spread across Europe with highest frequencies in the Near East (~11%)^33^, in south Europe (from ~4% to ~11%)^34^ and in the Balkan peninsula (~8%)^35^. HV0a coalesces around 12.5–11.0 kya ago^34^ and, among the extant populations, is common in Sardinia^36^.

Individual A, albeit at low coverage (Table 1), was found to belong to the Y-chromosome lineage A-M13 (A1b1b2b), a rare lineage absent among ancient individuals from the Italian Peninsula^29^, mainly found in Eastern Africa (~ 40%), but with known occurrences, at much lower frequencies, in the Near East (Turkey, Yemen, Egypt, Palestine, Jordan, Oman and Saudi Arabia) and the Mediterranean islands of Sardinia, Cyprus and Lesbos^37–40^. Downstream of A-M13 and restricting the analysis to transversions polymorphisms, the individual can be placed at A-V5880, a sub-haplogroup that contains all A-M13 positive Sardinians from past studies^39,40^, and that has been dated to coalesce around 7.62 (± 0.92) thousands of years ago, using Bayesian analysis^40^.

### Pompeian genetic structure

To understand the relationship of the higher-coverage ancient Pompeian individual A, we assembled a dataset of relevant previously published ancient populations from Upper Palaeolithic to Medieval periods^13–15,29,41–66^ (genotyped on the “1240K” SNP panel—detailed in Supplementary Table S2) combined with 471 present-day West Eurasian individuals (subset panel of the Human Origins)^56^, which was used on all subsequent analyses.

**Figure 2:**
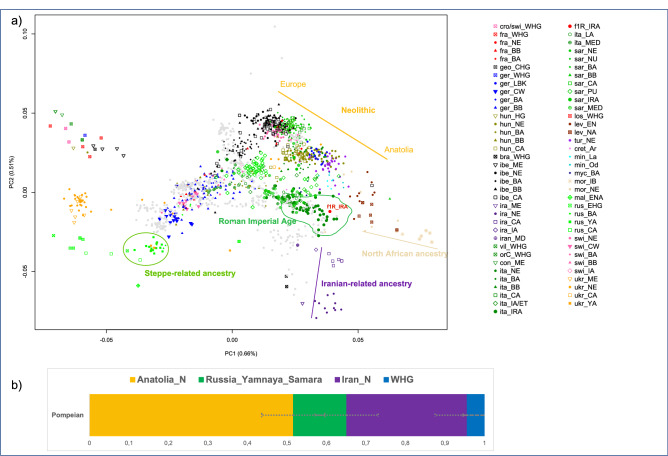
(**a**) PCA on selected 471 present-day west Eurasians (gray dots) and projected 1030 ancient individuals plotted by R version 3.6.2 (https://www.r-project.org/): the Pompeian individual A is in red and labelled with f1R_IRA; (**b**) four-way qpAdm models with source populations Anatolia Neolithic, Russian_Yamnaya_Samara, Iranian_N and Western hunter-gatherers (WHG). Error bars represent ± 1 standard errors of the proportion of each component. The complete results are reported in Supplementary Table S3.

Using the EIGENSOFT package^67^, we performed principal component analysis (PCA), and according to the results the Pompeian individual A clustered with other Italian Imperial Roman Age individuals and is positioned close to the well-documented Neolithic cline of Anatolians to European populations^58^ (Fig. 2a).

These results can be formally tested using a *D*-statistics of the form *D*(Mbuti, Test; Pompeian, Italy_IRA), which gauges whether the Pompeian individual A forms a clade with other Central Italian Imperial Roman Age individuals^29^, to the exclusion of other test populations (Supplementary Table S4). With the exception of Bronze Age Iberia, which shares a high genetic drift with Italy Imperial Roman Age (Z = 3.33), for all the other populations considered we cannot reject a clade-like relationship for the Pompeian and Imperial Roman Age cluster (Supplementary Table S4).

**Figure 3:**
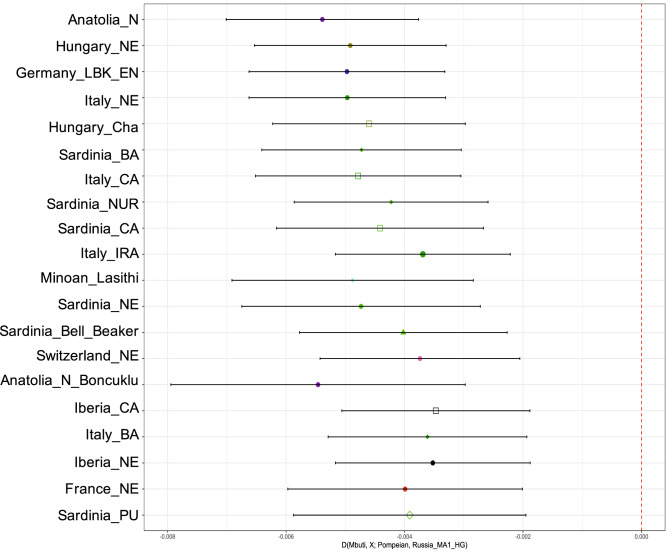
Point estimates and ± 3 standard errors for the top twenty populations with significantly (Z < 3) more allele sharing with the ancient Pompeian in comparison to 24 ky old Mal’ta based on the statistic D(Mbuti, Test; Pompeian, Russia_MA1_HG). All results can be found in Supplementary Table S5.

Furthermore, using statistics in the form *D*(Mbuti, Test; Pompeian, Russia_MA1_HG), we tested which other population showed high affinity to the Pompeian individual, using a 24 thousand year old individual from Russia as an outgroup (Mal’ta)^65^. Populations with Z-score values lower than − 3 represent statistically significant results of excess shared drift with Pompeii individual A. Among them, Neolithic Anatolian (Anatolia_N) had the highest scores, with Z = − 9.57 (Supplementary Table S5, Fig. 3).

The position of the Pompeian individual A in the PCA (Fig. 2a) falls also close to the distribution of modern Mediterranean and Near Eastern populations, such as Greeks, Maltese, Cypriots, and Turks. Such a result allowed us to hypothesize a genetic contribution from the Near East. This hypothesis can be also supported by a cline from Neolithic Iran (Iran_N) to Italy Imperial Roman Age (Italy_IRA, including the Pompeian individual) passing through Chalcolithic Iran (Iran_CA) and Iron Age Iran (Iran_IA) (Fig. 2a). The presence of Iranian-related ancestry has been identified in Italy since the Neolithic period, with a reported increase in this component in Central Italy during the Roman Imperial Age compared to the previous Iron Age period^29,66^. However, when performing the same four-population test, but using the Pompeian individual instead of Imperial Age Romans from Rome, the result is statistically non-significant (Supplementary Table S6), indicating that in individual A of Pompeii no further contribution by Iranian-related ancestry occurred after the Iron Age.

From the distribution of individuals obtained with our PCA analysis (Fig. 2a), it is also possible to recognize a cline from Morocco_Iberomaurusian to Italy Imperial Roman Age (Italy_IRA) passing through Morocco Neolithic after the Iron Age. The genetic contribution derived from a North African source is already evident in the Italian prehistory. Indeed, admixture of a North African ancestry was recognized in Sardinia since the Chalcolithic^48^ and in central Italy since the Iron Age (Etruscan)^29,66^ and continued into the Roman Imperial period^29^. Nevertheless, we did not identify any North African ancestry contribution in the Pompeian individual using *D-*statistics (Supplementary Table S6). Another significant genetic component in most post-Bronze Age European populations comes from a source ultimately deriving from the Eurasian Steppe^41,68^, and has been attested in the Italian Peninsula in Iron Age Italy^29,69^, Bronze Age Sicily^45^ as well as in the Pompeian individual A.

To confirm these findings, we attempted to fit the Pompeian as either a three- or four-way combination of Anatolian Neolithic, Russian Yanmaya, Iranian Neolithic and Western Hunter-Gatherers (WHG) using *qpAdm*^70^ (Supplementary Table S3). We set a minimum threshold of 100,000 SNPs and only considered results when p > 0.05.

Three-way admixtures that fit the data always included both ancestries from Anatolian and Iranian Neolithic, with varying contributions from the Steppe and WHG (Supplementary Table S3). Four-way models displayed major contributions of Anatolian Neolithic (51.6 ± 7.8%) and Iran Neolithic (30.5 ± 8.1%) in comparison to Steppe-related ancestry (13.5 ± 8.0%) and WHG (4.4 ± 5.4%) components (Fig. 2b, Supplementary Table S3).

We further attempted to investigate if the fit could be improved by including Morocco_Iberomaurusian as a fifth source^46^, but no three-, four- or five-way mixture with this last component produced significant results (Supplementary Table S3).

### Mycobacterium tuberculosis

**Figure 4:**
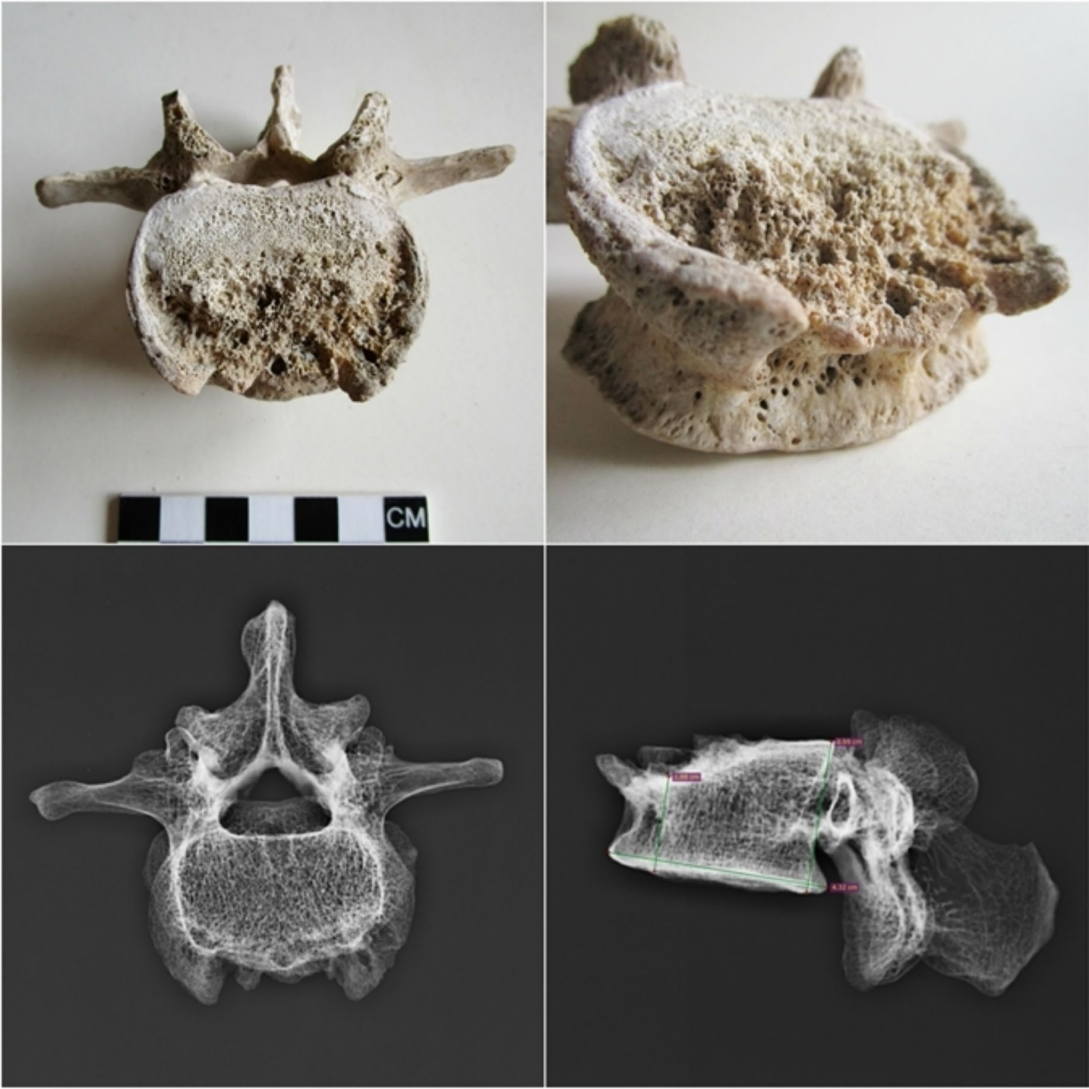
Photography and digital radiograph of the fourth lumbar vertebra (L4) affected by tuberculous spondylodiscitis of the individual A.

A palaeopathological study carried out on the Pompeian individual diagnosed spinal tuberculosis (Pott's disease) (Table 2) on the basis of diagnostic morphological markers such as a large lytic destruction on the upper anterior half of the fourth lumbar vertebra L4 (Fig. 4). Moreover, the digital radiograph analysis shows erosion in the antero-superior portion of the vertebral body, with a reduced downward cortical rim and a bowl-shaped appearance (Fig. 4). All the other vertebral osteitis that can cause similar lesions (pyogenic osteomyelitis, actinomycosis, metastatic neoplasms, osteoporosis, brucellosis and extrapulmonary tuberculosis^71^) have been excluded for the following reasons.

**Table 2:** Differential diagnosis of tuberculosis in individual A from “Casa del Fabbro”. nd: characteristic not determinable.

Lesion	Individual A	Actinomycosis	Tuberculosis	Brucellosis	Osteoporosis	Metastatic neoplasia	Pyogenic osteomyelitis
Spinous process and neural arch are involved^71,73^	No	No	No	No	Yes	Yes	Yes
Large and spheroid lesions surrounded by reactive new bone^72^	No	Yes	No	No	No	No	No
First affects the peduncles^70^	No	No	No	No	No	Yes	No
Vertebral fracture^73^	No	No	Possible	No	Yes	No	No
Demineralization^73^	No	No	No	No	Yes	No	No
Vertebral bodies in lumbar tract affected^71,73,74^	Yes	No	Yes	Yes	Yes	No	Yes
Collapse of vertebral bodies and angular deformity^74^	Yes	No	Yes	Yes	No	No	No
At least two adjacent vertebrae affected in most of cases^71^	nd	No	Yes	No	No	No	Yes
Sign of Pedro-i-Pons^74^	No	No	No	Yes	No	No	No

Pyogenic osteomyelitis may cause lytic lesions of vertebral bodies, with a predilection for the lumbar spine; however, the spinous process and neural arch are often involved, and lesions often show marked new bone proliferation^71^. Actinomycosis is characterized by large and spheroid lesions surrounded by reactive new bone; the vertebral column is affected in a way quite different from that of any other form of infection^72^. Metastatic neoplasia first affects the peduncles, neural arches and spinous processes with circular lytic lesions, and later the vertebral body. Osteoporosis, the most frequent demineralizing disease, can produce vertebral fracture and collapse due to bone demineralization^73^ and normally affects aged people. The brucellar spondylitis, a consequence of brucellosis, a highly contagious zoonosis caused by ingestion of milk or infected meat, has an evolution similar to Pott's disease (extrapulmonary tuberculosis), so much so as to be called pseudo-Pott. It involves destructive vertebral lesions and formation of ossifluent abscesses. Moreover, at digital radiograph analyses, brucellar spondylitis shows disseminated decalcification, irregular erosions at the vertebral edges with the typical destruction of the upper anterior angle of one or more contiguous vertebrae, reduction of the intervertebral space up to the fusion between two contiguous vertebrae and the Pedro-i-Pons sign^74^ (osteosclerotic semicircle around the osteolysis area of the upper antero-vertebral angle, typical of brucellar spondylitis). The digital radiography image shows no sign of Pedro-i-Pons (Fig. 4), and the case fully matches the palaeopathological and radiological criteria for tuberculosis proposed by Buikstra and Roberts^74^.

Extrapulmonary tuberculosis can cause characteristic skeletal changes, such as collapse of the vertebrae (Pott's disease), periosteal reactive lesions, and osteomyelitis.

Accordingly, the most probable diagnosis for Individual A from *Casa del Fabbro* is spinal TB (Pott's disease) (Table 2), the most common type of tuberculosis involving the bony elements, and one of the most common and devastating diseases in human history^75^. The partial preservation of the lumbar spine does not allow us to observe if the lesions also affected the L3 which has not been recovered. Nevertheless, the degree of extension of the lesion on the upper body of L4 prompts us to think that the lower body of L3 was also probably affected. The importance of this case is also because skeletal injuries, the only events that can be identified in an archaeological context, occur only in a minority of cases. Pre-antibiotic data suggest that about 3–5% of TB cases show bone alterations^74^.

As it is known that the DNA from ancient pathogens can also be preserved on ancient bone material^76^, we attempted to recover *Mycobacterium tuberculosis* DNA from the sequenced reads of the individual palaeopathologically diagnosed with TB. For this, we used a k-mer-based competitive metagenomic classifier^77^ on a database containing all archaea, bacterial and viral genomes, as well as the human reference. This resulted in 14,096 reads ranked at the *Mycobacterium* genus, but only 403 of those could be uniquely assigned to the *Mycobacterium tuberculosis* species complex (Supplementary Table S7). We extracted and remapped those reads to *Mycobacterium tuberculosis* human strain H37Rv and evaluated deamination patterns. When using reads from the whole *Mycobacterium* genus, we indeed have a damage pattern consistent with aDNA (Supplementary Fig. S4), but when restricting to reads classified as *Mycobacterium tuberculosis* complex the low amount of data does not provide enough resolution for authentication. This result could be due to three different, not mutually exclusive, reasons: it is already known that the petrous bone is an excellent source of endogenous aDNA but it shows little aDNA from pathogens^76^; in addition, *Mycobacterium tuberculosis* is known to be difficult to molecularly diagnose even on affected and symptomatic patients from buccal swabs^77–80^. Moreover, within the *Mycobacterium* genus, the *Mycobacterium tuberculosis* shares up to ∼ 99% genetic sequence identity with other common soil Mycobacteria^81^. However, in our case, the two individuals had never been in contact with the soil during the diagenesis process, because they were entirely covered by volcanic material. This makes the finding of the *Mycobacterium tuberculosis* DNA more likely to be endogenous.

## Discussion

To our knowledge, our results represent the first successfully sequenced Pompeian human genome.

The genome-wide analyses point out that the Pompeian individual A is genetically close to the extant Mediterranean peoples, mostly to Central Italians and Sardinians. It is plausible to think that, thanks to the expansion and the increase in effective population size during the Roman Imperial Age^82^, the Roman genetic pool could have contributed to the nearby populations with a genetic signature that can still be recognized in the extant Mediterranean regions today. Consistent with the autosomal results that show a high affinity of individual A with Neolithic Anatolians, its Y-chromosome haplogroup is presently only found among present-day Sardinians^45^. This makes it likely that this male lineage arrived in the Italian peninsula through an Anatolian source during the Neolithic.

Both Y-chromosome and mtDNA lineages from the Pompeian individual were absent among published individuals in Roman Imperial age in Italy^29^, suggesting a high diversity during that period across the Italian Peninsula. This signal can also be seen at the genome-wide level, by comparing the estimated ancestry proportions found in individual A with those from published Roman Imperial Age individuals^29^ (Supplementary Table S8, Supplementary Fig. S5). Actually, for some of the published individuals, it was not possible to reach a fit with the model using the same source populations (Supplementary Table S8), suggesting a different genetic composition. This genetic variability found in the Roman Imperial Age could be also supported by contacts, interactions, and migrations of people across the Mediterranean basin already identified using different methodologies^83,84^.

Despite this high genetic variability in the Imperial period^29^ the Pompeian individual A shows a higher level of shared genetic drift with the central Italy Roman Imperial Age group. This result strongly suggests that the individual that we have analysed should come from the Italian peninsula. Whether this individual belongs to the local population of Pompeii or is part of the 5% of the internal migrants characterizing the imperial population of Italy^85^ is difficult to address, but very likely he is not part of the large external migrations related to the practice of enslavement.

Finally, we used multiple lines of evidence to determine that one of the individuals was affected by tuberculosis. It is already known that tuberculosis was endemic in the Roman Imperial period thanks to the writings and ancient descriptions from Celsus (De Medicina, III, 22; 1st cent. AD), Galen and Celio Aureliano, and Aretheus of Cappadocia (Signa chron., I, 8; 2nd cent. AD). The increased population density that characterized the beginning of the Roman era, probably due to the development of an urban Roman way of life, favoured the spread of tuberculosis across Italy^86–88^.

In conclusion, our study—albeit limited to one individual—confirms and demonstrates the possibility of applying palaeogenomic methods to study human remains from this unique site. Our initial findings provide a foundation to promote an intensive analysis of well-preserved Pompeian individuals. Supported by the enormous amount of archaeological information that has been collected in the past century for the city of Pompeii, their paleogenetic analyses will help us to reconstruct the lifestyle of this fascinating population of the Imperial Roman period.

## Materials and methods

### Skeletal biology

Sex-determination was carried out using DSP (Diagnose Sexuelle Probabiliste)^89^.

Age-at-death was determined using changes to the pubic symphysis^90^ and the auricular surface of the ilium^91^ for both individuals. Concerning individual A, a radiographic method based on the apposition of secondary dentine was applied to the digital radiograph of the two left lower premolars^22^. Periapical digital radiographs were taken using a NOMAD hand-held dental X-ray device (Aribex, USA) combined with a digital sensor (DSX, Anthos, Italy) linked to a portable pc. All radiographs were taken with a Rinn-type digital sensor holder with 0.05 s exposure time and 60 kV.

Stature was estimated using statistical methods, taking into account the maximum length of long bones (humerus, ulna, femur and tibia), according to Giannecchini and Moggi-Cecchi^22^, and by applying the equations proposed by Pearson^76^ and by Trotter and Gleser for African-American^19–21^ that provide the most consistent estimates for Italian populations of Roman Age^18^.

Moreover, Pott's disease, a form of osteo-articular tuberculosis (TB), was examined by morphological approach and digital radiograph of the fourth lumbar vertebra (L4). For radiographic images, a DR Fujifilm machine was used with an exposure (100 ms) at 55 kV to 100 mA.

### Ancient DNA

#### DNA extraction

In order to obtain the largest possible amount of aDNA^15^ we have sampled one petrous bone for each individual. The ancient DNA extraction was performed according to the Allentoft et al.^41^ protocol. All molecular work (DNA extraction and library preparation) was conducted in dedicated ancient DNA clean laboratory facilities at the Center of Molecular Anthropology for Ancient DNA Studies, Department of Biology, University of Rome Tor Vergata^4^. The otic capsule was targeted and around 100–200 mg bone powder was used for DNA extraction. In order to remove the surface contaminants, the samples were pre-digested using a digestion buffer (0.46 M of EDTA pH = 8; 10 nm of TE buffer 100×; 0.14–0.22 mg/ml of Proteinase K; 0.5% of *N*-laurylsarcosine; 1/1000 vol of Phenol red) for 45 min at 37 °C. After that step, the samples were centrifuged at 2000*g* for 2 min and the supernatant was discarded, new digestion buffer was added for a 24-h digestion at 37 °C. The samples were then centrifuged at 2000*g* for 5 min and the pellet was stored for later re-extraction. The aDNA extraction was performed on the digested solution using Silica powder-based DNA extraction protocol. To each digested sample, 100 μl silica suspension and 10 × volume of binding buffer (4.88 M GuHCl and 29.3% 2-propanol; 1/1000 vol. of phenol red; 24.88 mM of NaCl; 87.6 mM of Na Acetate; final pH = 4) was added and adjusted to pH 4 with 37% HCl. The solution was incubated for 1 h at room temperature after which the samples were centrifuged for 2 min at 2000*g* and the supernatant was discarded. The silica was re-suspended in 1 ml of binding buffer, transferred on a new 2 ml tube and the aDNA was washed using ice-cold ethanol. Finally, the DNA was eluted in 80 μl Quiagen EB buffer.

#### NGS library preparation

The blunt-end DNA libraries were built from around 20 µl on DNA extracts using Illumina specific adapters and NEBNext DNA Sample Pre Master Mix Set 2 (E6070) kit according to manufacturer’s instruction with few modifications. For each step, negative library controls were included. 25 μl reactions mix were incubated at 12 °C for 20 min and 37 °C for 15 min for the end-repair step. A purification step using Qiagen MinElute spin columns was performed and DNA was eluted in 17 μl EB buffer. In the ligation step, Illumina specific adapters were prepared according to Meyer and Kircher^92^ and added to 15 μl of purified DNA. For ligation NEB Quick Ligation module (E6056L) was followed and the mix was incubated at 20 °C for 15 min. Then, the mixture was purified using Qiagen MinElute spin columns and DNA was eluted in 20 μl EB buffer. In the last step, adapter fill-in reaction was performed incubating at 65 °C for 20 min and 80 °C for 20 min 30 μl of reaction mix. The quantification of the library was conducted using SYBER green mix according to manufacturer’s instructions and using IS8 and IS7 primers. The amount of DNA library was used to assess the optimal number of PCR cycles required for DNA library indexing. The indexing was performed on 20 μl DNA library using 2X Kapa U (following the manufacture’s temperature instruction) and 1 μl of each primer (10 mM, inPE forward primer and indexed reverse primer). The indexed amplified DNA libraries were then purified using Qiagen MinElute Kit and eluted in 50 μl EB buffer. To quantify the DNA libraries an Agilent Bioanalyzer 2100 was used, and the libraries were sequenced on Illumina HiSeq 2500 using v3 chemistry and paired end (PE) 100 cycles.

#### Bioinformatics and DNA authentication

To remove the Illumina adapter sequences, we used AdapterRemoval 1.5.2^93^. The adapter-free reads were then mapped against human reference genome built 37 using BWA 0.6.2^94^ and only the aligned sequences with mapping quality at least 30 were sorted using samtools 0.1.18^95^. PCR duplicate reads were removed by Picard MarkDuplicate http://broadinstitute.github.io/picard/. All mapping statistics for the analysed individuals are reported in Table 1.

The aDNA authentication was evaluated assessing the damage pattern of the DNA, estimating the contamination in X chromosome and in mtDNA. The damage is one of the most important characteristics of the aDNA and includes deamination at the 5′ end of the DNA leading to the typical C to T transition as well as G to A at the 3′ end. The damage evaluation was assessed using mapDamage 2.0^96^.

The X chromosome contamination was evaluated by ANGSD^97^ following the commands suggested by the authors. The mitochondrial contamination was evaluated with contamMix^98^. ContamMix reports the contamination rate with a Bayesian estimate of the posterior probability of the contamination proportion. The method is based on consensus mitochondrial sequences analysis by comparing the mtDNA reads of ancient individuals to the reconstructed consensus and 311 present day whole mitochondrial genomes (the possible contaminants). The analysis was restricted only to individual A due to the low mtDNA coverage shown by individual B. To reconstruct the consensus sequence, the option –doFasta of ANGSD package^97^ was used and each SNP was called only if it presents at least at 3× coverage. The mitochondrial genome mapping was performed against the revised Cambridge Reference Sequence (rCRS) using base and reads mapping quality > 30.

#### Sex determination

The sex determination was evaluated by calculating the R_Y_ parameter, applying the Skoglund et al.^26^ python script. The R_Y_ parameter represents the fraction of the total number of reads aligned with the Y chromosome (nγ) divided the total number of reads mapped with both sex chromosome (nγ and nγ): R_Y_ = ny/(n_X_ + n_Y_). A R_Y_ parameter value above 0.077 is consistent with male individuals while a value lower than 0.016 with female ones.

#### Uniparental genetic markers analysis

For the mtDNA analysis the mitochondrial consensus sequences have been used and haplogroup was assigned using the command line version of HaploGrep2^27,28^.

Y-chromosome haplogroup placement was carried out first using *PathPhynder*^99^, a software designed to place ancient and/or low-coverage individuals in a high-confidence phylogeny. While *PathPhynder* was able to fit individual A in the tree, the paucity of publicly available individuals from haplogroup A hindered a higher definition. We then proceeded to use ANGSD^97^ to call genotypes at all phylogenetically informative sites listed in ISOGG. This curated database was split into transition and transversion variants to allow the usage of different filters, taking into consideration ancient DNA damage that manifests itself mainly through transitions^25^. Only variants with over 95% frequency in the population of non-clonal reads in the loci were called. When calling transition variants, 7 bases were trimmed in each read end, following mapDamage 2.0^96^. Although haplogroup A1b1 is the sister branch of BT—a macro-haplogroup that contains all other Y-chromosome haplogroups other than A00, A0 and A1a—less than 50 SNPs are listed in ISOGG 2020 either defining or downstream to it. Therefore, we further included all SNPs listed in previous studies^39,40^ and from ISOGG raw data (https://ybrowse.org/gbrowse2/gff/) belonging to haplogroup A. Those variants were filtered to include exclusively single nucleotide point mutation biallelic variants located in the ~ 10 Mb short-read mappable region of the Y-chromosome^100^. Recurrent mutations of lower phylogenetic confidence were removed by checking whether any identical SNP (genomic position and derived base) was found downstream of BT. The haplogroup placement was further confirmed by checking whether the individual carried the ancestral alleles for downstream haplogroups BT, CT, CF, DE, F, GHIJK, IJ, P1 and NO. All SNP calls are reported in Supplementary Table S9–S13. Code and data for the Y-chromosome analyses are available at github.com/tpinotti/Pompeya.

#### PCA and model-based clustering

To assess the genetic relationship between the Pompeian individual A and other ancient/modern populations, we merged the whole-genome shotgun data generated in this study with published datasets of modern and ancient populations by using Plink (www.cog-genomics.org/plink/1.9/)^101^. A subset panel of the Human Origins panel published by Lazaridis and colleagues^56^ based on modern Western Eurasian populations (Albanians, Ashkenazi, Basques, Belarusians, British, Bulgarians, Croatians, Cypriots, Czechs, Estonians, Finns, the French, Greeks, Hungarians, Icelanders, Italians, Lithuanians, Maltese, Mordovians, Norwegians, Orcadians, Russians, Sardinians, Scottish, Spanish, Turks, Ukrainians), and selected 1030 published ancient individuals^13–15,29,41–66^ was used (Supplementary Table S2). We performed a Principal Component Analysis (PCA) using the EIGENSTRAT method^102^ included in the EIGENSOFT package^67^, projecting the ancient individuals onto the components calculated for modern Western Eurasian populations using “lsqproject” and “shrinkmode” options of smartpca.

#### D- and f-statistics and population differentiation

To investigate patterns of shared ancestry in our dataset, *D*-statistics analyses were performed using the Admixtools 7.0 package^70^. Only values of *D*-statistics for which |Z|> 3 were considered as statistically significant. The individuals were grouped in the same population according to the cultural and period information reported to the Supplementary Table S2 (column: Label for analyses).

#### qpAdm

We performed *qpAdm* on the pseudo-haploid genotype of the Pompeian individual using *qpAdm* v7.0^70^ applying the option “allsnps: YES”. A set of 15 outgroups was used as "right populations": Mbuti, UstIshim, Caucasian hunter-gatherers (CHG), Eastern hunter-gatherers (EHG), Villabruna, Russia_MA1_HG, Natufian, Jordan_PPNB. We tested several models, and we only plotted the model with p > 0.05. Because we are modelling a single individual, we set a minimum threshold of 100,000 SNPs^45^.

Using the same parameters we also performed the *qpAdm* analysis on already published Roman Imperial Age individuals^29^ (Supplementary Table S8) and we only plotted the models with the higher p-value (p > 0.05) where the inferred admixture proportions were also inside the interval [0, 1] (Supplementary Fig. S5).

#### Pathogen screening

We used Kraken2^77^, a k-mer-based competitive metagenomic classifier, to find matches on a database of all archaea, bacterial and viral genomes, as well as the human reference, with custom parameters (-confidence 0.05 -minimum-map-quality 30). 3.90% of reads were classified as Bacteria, and 14,096 of those were ranked at the *Mycobacterium* genus (Supplementary Table S7). However, only 403 reads could be uniquely assigned to the *Mycobacterium tuberculosis* species complex (Supplementary Table S7). We extracted those reads and mapped those to *Mycobacterium tuberculosis* human strain H37Rv using bowtie2^103^ using custom parameters (-D 20 -R 3 -N 1 -L 20 -i S,1,0.50 -end-to-end^104^) obtaining 161 and 45 unique reads, respectively (Supplementary Table S7). While these reads could stem from actual circulating *Mycobacterium tuberculosis* found in the individual, the low amount of data does not provide enough resolution to authenticate or to perform downstream analysis on it.

## Supplementary Information

Supplementary Tables.

Supplementary Figures.

## Data Availability

The allignment bam file generated in this study has been deposited in the Zenodo database under the permanent DOI: 10.5281/zenodo.6468368.
