# Supplementary material for: Bioarchaeological and palaeogenomic portrait of two Pompeians that died during the eruption of Vesuvius in 79 AD
Source: Sci Rep. 2022 May 26;12:6468. doi: 10.1038/s41598-022-10899-1 (PMC9135728; doi:10.1038/s41598-022-10899-1)
Supplement: Supplementary file 2 — Supplementary Figures. [file 41598_2022_10899_MOESM2_ESM.docx]

**Bioarchaeological and palaeogenomic portrait of two Pompeians that died during the eruption of Vesuvius in 79 AD:**

**Supplementary Material**

Gabriele Scorrano^1, 2, *^, Serena Viva ^3^, Thomaz Pinotti^2,4^, Pier Francesco Fabbri ^3, *^, Olga Rickards ^1, !^, Fabio Macciardi ^5, !, *^

^1^ Centre of Molecular Anthropology for Ancient DNA Studies, Department of Biology, University of Rome “Tor Vergata”, Rome, 00133, Italy.

^2^ Lundbeck Foundation GeoGenetics Centre, Globe Institute, University of Copenhagen, Copenhagen, Denmark.

^3^ Department of Cultural Heritage, University of Salento, Lecce, 73100, Italy

^4^ Laboratório de Biodiversidade e Evolução Molecular (LBEM), Universidade Federal de Minas Gerais, Belo Horizonte, Brazil.

^5^ Laboratory of Molecular Psychiatry, Department of Psychiatry and Human Behavior, University of California, Irvine, 92868, CA, USA.

^!^ These authors contributed equally.

^*^ Correspondence: Gabriele Scorrano: gabrielescor@gmail.com, g.scorrano@sund.ku.dk; Pier Francesco Fabbri pierfrancesco.fabbri@unisalento.it; Fabio Macciardi: fmacciar@uci.edu

**Supplementary Fig. S1**: Bone remains pertinent to the individual A and the individual B.

**Supplementary Fig. S2**: Patterns of aDNA damage in the analysed individuals: in red C to T, in blue G to A for the individual A (a) and B (b).

**Supplementary Fig. S3**: mitochondrial contamination test result (contamMix output) of individual A.

**Supplementary Fig. S4**: Damage pattern in red C to T in blue G to A for the *Mycobacterium* genus alignment (see also Supplementary Table 7).

**Supplementary Fig. S5**: three-to-five-way *qpAdm* models with source populations: Anatolia Neolithic, Morocco_Iberomaurusian, Russian_Yamnaya_Samara, Iranian_N and Western hunter-gatherers (WHG). Error bars represent standard error of the proportion of each component. The complete results are reported in Supplementary Table S8.
